# The efficacy and safety of Retcam in detecting neonatal retinal hemorrhages

**DOI:** 10.1186/s12886-018-0887-y

**Published:** 2018-08-20

**Authors:** Feng Chen, Dan Cheng, Jiandong Pan, Chongbin Huang, Xingxing Cai, Zhongxu Tian, Fan Lu, Lijun Shen

**Affiliations:** 1 School of Optometry and Ophthalmology and Eye Hospital of Wenzhou Medical University, Number 270, West Xueyuan Road, Lucheng District, Wenzhou, 325000 Zhejiang China; 2 Neonate Department, Yueqing Maternal and Child Health Hospital, Yueqing, China

**Keywords:** Neonatal retinal hemorrhage, Digital imaging, RetCam, Fundus examination, Systemic effects

## Abstract

**Background:**

To investigate the ability of characterizing neonatal retinal hemorrhage (RH) using RetCam in healthy newborns and the systemic effects during the procedure.

**Methods:**

This prospective study enrolled 68 healthy newborns aged 2 to 4 days old. The RH was imaged and classified according to the location and numbers of hemorrhages. The heart rate (HR), respiration rate (RR), and oxygen saturation (OS) were recorded at 4 time points before (Phase 1, P1), during (P2 and P3) and after the examination (P4).

**Results:**

The median exam time was 151 s. RH was present in 15 infants and 23 eyes. All 23 eyes had hemorrhage in Zone II. Grade II and III hemorrhages were present in 5 and 18 eyes, respectively. The HR increased to 168 beats per minute (bpm) in P3 and recovered to 122.5 bpm in P4. The RR increased to 38 bpm in P3 and recovered to 25 bpm in P4. The OS was reduced to 83% in P2 and recovered to 96% in P4.

**Conclusions:**

RH in healthy newborns, mostly present in Zone II with grade II and III, can be characterized in detail by RetCam. Systemic effects during the process are mild and can be revolved spontaneously.

## Background

With its initial detection in 1861, retinal hemorrhage (RH) in newborns has been reported frequently [1]. The incidence of neonatal RH (NRH) varies widely from 2.6 to 50.0% [2–8]. Birth-related RH is commonly bilateral, intraretinal, localized primarily to the posterior retina, and rapidly resolved without any visual deficits [1, 9, 10]. The follow-up of the development of birth-related hemorrhage may play a significant role in the process of neonatal eye examination [11]. In addition, an accurate description of retinal bleeding is extremely important given its association with abusive head trauma [1, 12]. The direct ophthalmoscope and indirect ophthalmoscope were successively used for the primary examination of the fundus of the newborns. Nevertheless, the examination results had inadequate examination range or were particularly subjective. Currently, a wide-angle fundus camera (RetCam, Clarity Medical Systems USA) allowing immediate visualization and real-time recording of fundus findings has become widely used in fundus examination and has potential in recording RH on newborns [13, 14].

To our knowledge, the systemic effects of the fundus examination in healthy newborns have not been studied. Several studies have reported on systemic effects of the screening examination on retinopathy of prematurity (ROP) in preterm infants [15–20]. The preterm infants exhibited increased blood pressure, decreased oxygen saturation, increased pulse rate [15], CRIES pain score [17], facial responses to pain [18], and salivary cortisol [19] after examination. The healthy newborns’ behavior during the fundus examination may differ from the preterm infants’ behavior based on the existence of different demographics. This study aimed to detect the ability of screening birth-related RH by RetCam examination and to identify any significant systemic effects during the process in healthy newborns.

## Methods

This study was approved by the research ethics committees at Eye Hospital of Wenzhou Medical University (Wenzhou, Zhenjiang, China), and informed parental consent was obtained before the study. This study adhered to the tenets of the Declaration of Helsinki. Consecutive 68 healthy newborns receiving neonatal RH screening examination during a two-week study period at Yueqing Maternal and Child Health Hospital (Yueqing, Zhejiang, China) were included in the study from January 2013 to February 2015. Babies with known or suspected systemic or ocular disease or congenital malformation were excluded.

The fundus examination was performed by an experienced ophthalmologist with a digital wide-angle retinal imaging device (RetCam III; Clarity Medical Systems, Pleasanton, California) within 3 days after the birth of newborns. A neonatologist was always on standby during the examination to manage the subjects’ systemic condition. Every baby was offered a soother to suck during the examination unless it rejected the sucker. Pupillary dilatation was obtained with 0.5% tropicamide phenylephrine eye drops (Santen Pharmaceutical Co., Osaka, Japan) instilled twice every 5 min one hour before examination. Immediately before the eye examination, local anesthetic eye drops were administered with 0.5% proxymetacaine (Alcon Laboratories, Texas, USA). The eyelid was opened using an infant speculum (MR-0103-1, Xiehemedical, Suzhou, China). The 130 diopter camera lens was placed on the cornea after carbomer eye drops (Bausch & Lomb, Berlin, Germany) and applied onto the cornea. Both eyes of every newborn were examined, and there was an approximately 10-s pause between the time when the camera lens switched from the right eye to left eye. For each eye, 5 images were obtained that covered the posterior pole, temporal quadrant, superior quadrant, nasal quadrant and inferior quadrant, separately. By pushing the eye globe with the camera lens, the peripheral image was obtained. Fundus examination was performed in the morning between 9:00 and 12:00 AM, and the infants kept in incubators. Newborns with RH were re-examined 4 weeks later.

**Fig. 1:**
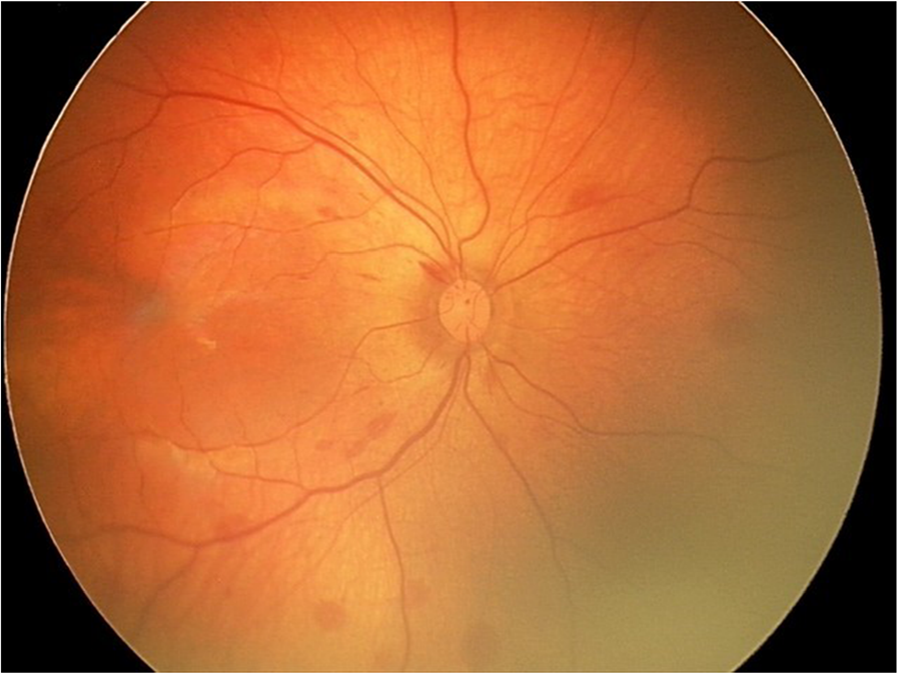
Retcam photograph of a grade II retinal hemorrhage in a newborn

**Fig. 2:**
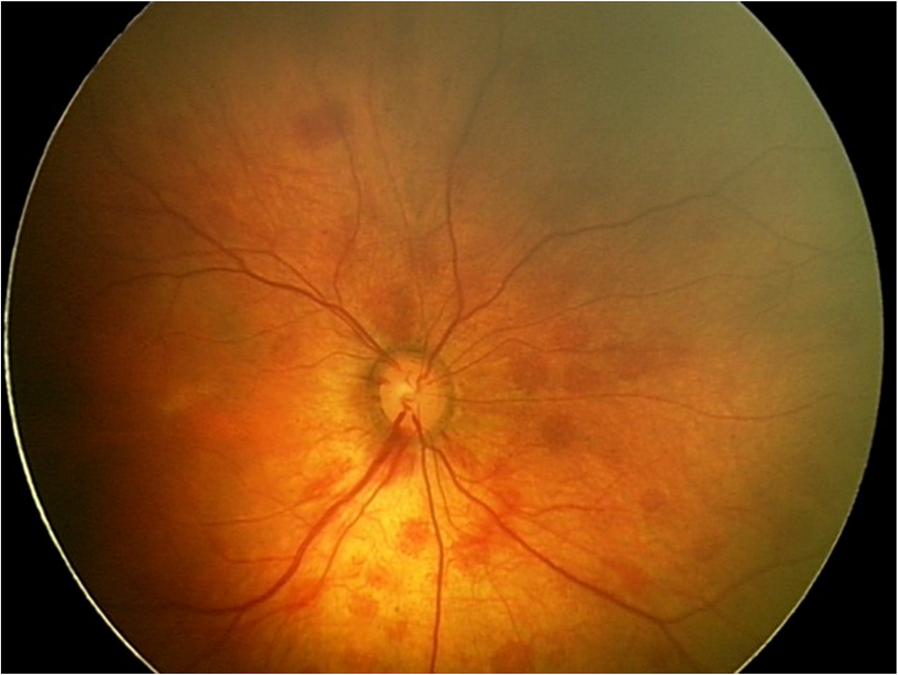
Retcam photograph of a grade III retinal hemorrhage in a newborn

Hemorrhages were classified according to the location and number of the hemorrhages by two masked readers (FC and JP) after the examination [1]. Zone I encompassed one disc diameter around the optic nerve head and fovea. Zone II extended from the anterior boundary of zone I to the equator. Zone III was anterior to zone II, extending to the ora serrata. Hemorrhage grade was determined by the number of hemorrhages. One or two hemorrhages were defined as grade I, three to ten hemorrhages grade II (Fig. 1), and more than ten hemorrhages grade III (Fig. 2).

Heart rate (HR), respiration rate (RR) and oxygen saturation (OS) were monitored by a monitor (Mindray patient monitor, model PM-8000 Express, Shenzhen, China) during the procedure. These measurements were recorded at 4 time points before, during or after the examination. Phase 1 (P1, baseline) was at 5 min before the eye examination. Phase 2 (P2) was when the camera lens was placed on the first eye’s cornea. Phase 3 (P3) was when the camera lens was switched to the second eye. Phase 4 (P4) was at 10 min after the procedure. Adverse systemic effects were monitored for during and short-term after the exam, including bradycardia (oculocardiac reflex, defined as percentage decrease in HR from baseline of ≥10%) and oxygen desaturation during examination, which was defined as a decrease in SaO2 of ≥20%.

The analyses were performed with SPSS software version 22.0 for Windows (SPSS Inc., Chicago, IL, USA). Given that data were not normally distributed, nonparametric statistics (2 related samples, Wilcoxon Signed Ranks Test) was performed to assess the changes in heart rate, respiration and oxygen saturation compared with baseline. The median and range was used to describe each variable. A level of *P* < 0.05 was accepted as statistically significant.

## Results

Demographic features of the newborn population are provided in Table 1. The subjects included 38 males and 30 females, all of which were full-term (born at 37 weeks or older). Fifty newborns were delivered by spontaneous vaginal delivery, and eighteen were delivered by cesarean section.

**Table 1:** Clinical Data for the Included Newborns (*n* = 68)

	Age (days)	Gestational age (weeks)	Birth weight (g)	1-minute Apgar score	5-minute Apgar score
Median	3	39.0	3300	9	10
Range	1–4	37.0–41.0	2150–4300	7–9	9–10

The median time for each newborn’s fundus examination using RetCam was 151 s (range 101–273). All hemorrhages found in 15 (22%) subjects and in 23 (17%) eyes (Table 2) were intra-retinal. Of the 15 newborns with hemorrhage, 8 (53%) had hemorrhage in both eyes, and 7 (47%) had hemorrhage in one eye. Hemorrhages were dot blot or flame shaped. Nineteen (14%) eyes had hemorrhages in Zone I, and 10 (7%) eyes had hemorrhages in zone III. All 23 eyes had hemorrhage in zone II. All the hemorrhages in this study were grade II or III, which were present in 5 (4%) and 18(13%) eyes, respectively. At the follow-up time, which was 4 weeks after birth, all the RHs disappeared completely.

**Table 2:** Characteristics of Retinal Hemorrhage in Healthy Newborns (*n* = 136)

	Zone			Grade		
	I	II	III	I	II	III
Eyes (n = 136, %)	19 (14%)	23 (17%)	10 (7%)	0	5 (4%)	18 (13%)

**Fig. 3:**
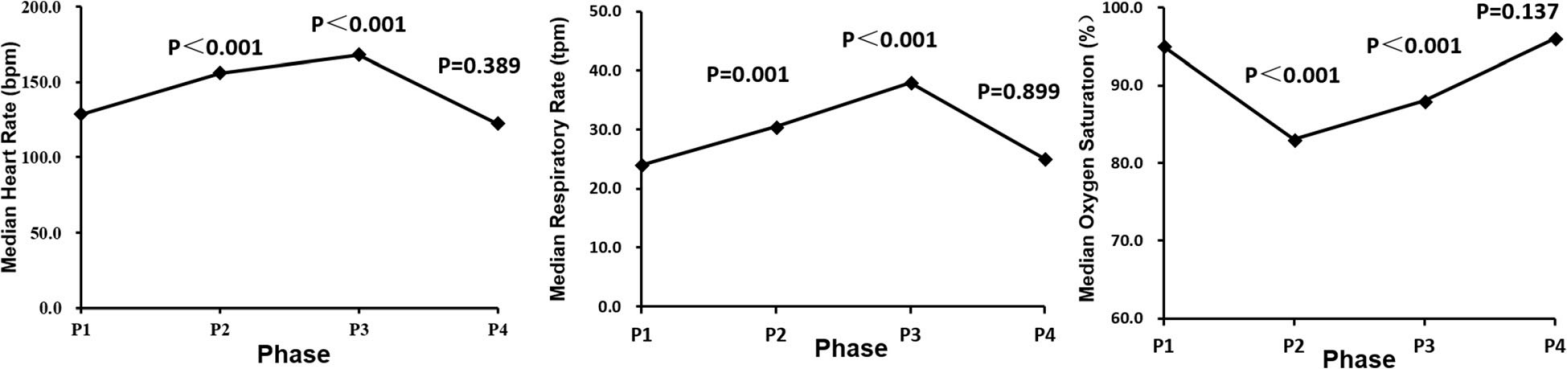
Change of heart rate (HR), respiratory rate (RR) and oxygen saturation (OS) during fundus examination with Retcam. HR and RR increased, OS decreased during the exam but recovered at 10 min after the exam. Values are medians. Phase 1 (P1, baseline) was at 5 min before the eye examination. Phase 2 (P2) was when the camera lens was just placed on the cornea of the first eye. Phase 3 (P3) was when the camera lens was switched to the second eye. Phase 4 (P4) was at 10 min after the procedure.* *P* < 0.05 compared to P1

At baseline, the median heart rate of the 68 newborns was 128.5 beats per minute (bpm, range 87–174). The heart rate increased to median value of 156 and 168 bpm in P2 and P3, respectively, but recovered to 122.5 bpm in P4 (Table 3, Fig. 3). Respiratory rate increased from 24 bpm in P1 to 30.5 and 38 bpm in P2 and P3, respectively, and recovered to 25 bpm in P4. Oxygen saturation levels declined from 95 to 83% during the exam and then recovered to 88% and 96% in P3 and P4, respectively.

**Table 3:** Stress Responses to the Retcam Screening Examination

	P 1	P2	P 3	P 4
HR, bpm, median (range)	128.5 (87–174)	156** (78–201)	168** (62–204)	122.5 (90–156)
RR, bpm, median (range)	24 (12–53)	30.5** (18–80)	38** (16–89)	25 (11–57)
OS (%), median (range)	95% (81–100%)	83%** (54–98%)	88%** (51–100%)	96% (82–100%)

*P1–4* Phase 1–4, *HR* Heart Rate, *RR* Respiratory Rate, *OS* Oxygen Saturation

**P ≤ 0.001 compared to P1, Sign Test

One subject (1.5%) developed bradycardia whose heart rate declined from 112 at baseline to 62 bpm during exam and subsequently recovered to 121 bpm at 10 min after the exam. Five subjects (7%) developed oxygen desaturation during examination in this study. The median of oxygen saturation of those 5 subjects declined from 97% (range 92–99%) at baseline to 70% (range 54–76%) during examination but recovered to 96% (range 95–98%) in P4.

## Discussion

The results of this study suggest that neonatal RH can be efficiently detected and characterized with digital imaging by RetCam. Healthy newborns have some systemic effects to RetCam examination; however, they can recover quickly after the examination. The systemic effects encompass the decrease in the heart rate and oxygen saturation.

Our study used RetCam imaging to record the morphology of RH in newborns. Previous studies have reported clinical and demographic features of infants with birth-related RH [1, 11, 21, 22]. To our knowledge, studies have rarely analyzed all the clinical grades of the hemorrhages [1, 23] in healthy newborns with RH by RetCam. RetCam imaging is an efficient tool to analyze the clinical classification of RH. The system causes minimal stress-related responses and provides rapid recording of fundus findings by images [13, 14], which may contribute to the security of neonatal screening and facilitate in clinical classification and follow-up examination. However, the incidence of the neonatal RH in this study is lower than values given in several other studies [2, 3, 5–7]. In the current study, 15 infants out of 68 didn’t develop RH. One important factor may be the mode of delivery. In this study, most newborns were delivered by spontaneous vaginal delivery, and a few were delivered by cesarean section. These two delivery modes tend to cause less incidence of NRH compared with vacuum-assisted delivery. The occurrence of NRH related to delivery by vacuum extraction was 75% in Emerson’s study [1] and 77.8% in Hughes’ study [24]. The high incidence of hemorrhage in babies born from vacuum-assisted vaginal delivery suggests that the neonatal RH is mainly caused by the change of pressure during the birth procedure. On the other hand, the variation of the incidence of NRH appears to be due to the age of the infants examined after birth and the mode of delivery. The median age of the newborns at examination was 3 days old in our study, which was older than those in other studies. The birth-related neonatal RH appears to resolve quickly, and the incidence declines as time progresses. Giles et al. reported that incidence of RH was reduced from 40% at 1 h post-delivery to 20% at 72 h [25].

The systemic response in infants screened for RH with techniques of examination should be taken into account to pediatricians and ophthalmologists. Previous studies demonstrated that a comprehensive evaluation of parameters standing for systemic response have been investigated, including blood pressure, CRIES pain score, facial responses to pain, and salivary cortisol. However, it was not possible to obtain blood pressure reading during the examination without interfering with its progress. Moreover, though ROP screening is considered a painful procedure, it may be different in the examination of healthy newborns and with digital imagings. The heart rate, respiration rate and oxygen saturation, which can be monitored simultaneously during the procedure with seldom interfering are evaluated in the current study. Oculocardiac reflex and oxygen desaturation are two complications during the fundus examination on newborns or premature infants. To the best of our knowledge, no studies have investigated systemic effects in healthy newborns during the detection of neonatal RH by RetCam. In this study, oculocardiac reflex was defined as a percentage decrease in HR from baseline of ≥10%, which is similar to other studies [14, 15, 26]. Clarke et al. reported that 17 out of 54 consecutive premature infants (31%) had oculocardiac reflex during indirect ophthalmoscopy examination [26]. The high incidence of oculocardiac reflex in their study may be associated with the method used. They depressed the sclera to detect the peripheral fundus in every infant. Mukherjee et al. reported that 8 (11.9%) in the RetCam group and 3 (8.3%) in the binocular indirect ophthalmoscopy (BIO) group developed oculocardiac reflex during ROP screening examination [14]. In their practice, a scleral depressor was used to gently rotate the globe not depress the sclera directly to obtain adequate visualization of the peripheral fundus, thus causing less oculocardiac reflex. Compared with those studies, the occurrence of oculocardiac reflex in our study is considerably reduced. One implication is that RetCam has a wider field of view than BIO, and thus globe rotation required is less than that of BIO. Another implication is that healthy newborns may behave different from premature infants during the fundus examination.

The incidence of oxygen desaturation during examination was 6% (5/80) in this study, which was similar to Laws et al.’s report [15]. The 5 subjects who developed oxygen desaturation did not suffer apneic episode, and the oxygen saturation returned to baseline levels rapidly without supplemental oxygen administration. Mehta et al. reported a much higher incidence of episodes of desaturation (9/42) in a small cohort of 12 neonates screened with BIO or RetCam [18]. They also found a greater incidence of episodes of desaturation with the RetCam and suspected that the longer time required for screening with the RetCam 120 might be a contributing factor to the difference. Mukherjee et al. did not identify any significant difference in episodes of desaturation associated with RetCam use [14]. In our opinion, the subjects’ gestational age at birth and postconceptional age at examination might account more for the occurrence of episodes of desaturation compared with the duration of the examination. In Mehta et al.’s study, the median gestational age at birth of the infants was 28 weeks, and the median postconceptional age at the first screening was 33 weeks. Younger infants may be more likely to develop oxygen desaturation during medical intervention.

Neonatal care may play an important role in the newborns’ response to medical intervention. A Newborn Individualized Developmental Care and Assessment Program (NIDCAP)-based intervention has been adopted by Kleberg et al. during eye examination for ROP [19]. In that study, the NIDCAP-based intervention did not decrease pain responses but resulted in faster recovery, as measured by lower salivary cortisol. NIDCAP is an intervention program aiming at optimizing and adapting neonatal care for preterm infants. NIDCAP included individual evaluation of the infant’s responses, direct support to the infant, pacing of the procedure, and modification of the environment. In the present study, we adopted some developmental care strategies that were in accordance with NIDCAP care guidelines. The examination environment was quiet and calm, and room lighting was moderate. During the examination, newborns were lying supine and wrapped with legs cocooned. A neonatologist was on standby, providing effective and individual support throughout the examination. There was a ten-second rest between the first and second eye. The above strategies seemed to minimize the newborns’ systemic effects in this study.

RetCam screening have several advantages over BIO in terms of efficacy and safety in neonatal eye examination. First, the classical ophthalmoscope are subjective and leave no records, while Retcam screening has digital imaging records. Moreover, the RetCam has a wider field of view than BIO, which may has an inadequate examination rage. Therefore, the globe rotation required in the RetCam exam is less than that required for BIO. Scleral depression, which is compulsory with BIO, is not necessary with RetCam. Further, the carbomer eye drops used with RetCam will moisten the subject’s cornea and avoid the sense of burning. Additionally, the level of illumination appears to be reduced with RetCam compared with BIO. Mehta et al. and Mukherjee et al. reported that the RetCam group examination time was significantly longer than that of BIO (14.5 min versus 9 min and 7.8 min versus 3.9 min, respectively) [14, 18]. In our study, the median time for each newborn’s fundus examination using RetCam was 151 s (2.5 min), which was considerably reduced compared with other studies. The difference may be partly due to the different subjects and different familiarity with the examination procedure.

The examination technique is another important factor accounting for the subject’s systemic effects. Several studies have compared the impact of retinopathy of prematurity (ROP) screening examination between a digital fundus camera and conventional BIO on systemic effects [14, 18, 20].Mehta et al. found that screening with the RetCam 120 and the BIO with a speculum caused a greater change in pulse and mean blood pressure and an increase in facial responses to pain during and immediately after screening compared with the BIO without the speculum [18]. Mukherjee et al. reported that screening for ROP with a digital fundus camera was associated with a significantly reduced stress-related response compared with conventional indirect BIO [14].

The limitations of this study include the small sample size. Though neonatal eye examination has the goal of discovering serious congenital, hereditary and acquired eye diseases in the neonatal period of heathy newborns, this procedure is still optional for the families in China. Furthermore, it is understandable that parents are not sure about the necessity of the neonatal screening and worry about the security during the examination. Second, the current study included no identification of long-term side effects of examination. Future studies will include larger sample sizes and longer follow-up time to completely understand the long-term adverse effects of the exams.

The current study characterized the appearance, location and grades of neonatal RH using RetCam imaging. Transient systemic effects were presented during the screening process, and their recovery after the examination demonstrated the security of RetCam imaging. Our study indicates that RetCam is an efficient and secure screening tool in detecting birth-related RH.

## Conclusions

RH in healthy newborns, mostly present in Zone II with grade II and III, can be characterized in detail by RetCam. Systemic effects during the process are mild and can be revolved spontaneously.

## Abbreviations

BIO: Binocular indirect ophthalmoscopy
bpm: Beats per minute
HR: Heart rate
NIDCAP: Newborn Individualized Developmental Care and Assessment Program
OS: Oxygen saturation
RH: Retinal hemorrhage
ROP: Retinopathy of prematurity
RR: Respiration rate
